# Cell Cycle-Dependent Flagellar Disassembly in a Firebug Trypanosomatid *Leptomonas pyrrhocoris*

**DOI:** 10.1128/mBio.02424-19

**Published:** 2019-11-26

**Authors:** Cynthia Y. He, Adarsh Singh, Vyacheslav Yurchenko

**Affiliations:** a Department of Biological Sciences, Center for BioImaging Sciences, National University of Singapore, Singapore, Singapore; b Department of Biotechnology, Indian Institutes of Technology, Kharagpur, West Bengal, India; c Life Science Research Centre, Faculty of Science, University of Ostrava, Ostrava, Czech Republic; d Martsinovsky Institute of Medical Parasitology, Tropical and Vector Borne Diseases, Sechenov University, Moscow, Russia; Yale University School of Public Health

**Keywords:** flagellar length regulation, *Leptomonas pyrrhocoris*, trypanosomatids, flagellum disassembly, flagellar length regulation

## Abstract

Current understanding of flagellum/cilium length regulation focuses on a few model organisms with flagella of uniform length. Leptomonas pyrrhocoris is a monoxenous trypanosomatid parasite of firebugs. When cultivated *in vitro*, L. pyrrhocoris duplicates every 4.2 ± 0.2 h, representing the shortest doubling time reported for trypanosomatids so far. Each *L. pyrrhocoris* cell starts its cell cycle with a single flagellum. A new flagellum is assembled *de novo*, while the old flagellum persists throughout the cell cycle. The flagella in an asynchronous *L. pyrrhocoris* population exhibited a vast length variation of ∼3 to 24 μm, casting doubt on the presence of a length regulation mechanism based on a single balance point between the assembly and disassembly rate in these cells. Through imaging of live *L. pyrrhocoris* cells, a rapid, partial disassembly of the existing, old flagellum is observed upon, if not prior to, the initial assembly of a new flagellum. Mathematical modeling demonstrated an inverse correlation between the flagellar growth rate and flagellar length and inferred the presence of distinct, cell cycle-dependent disassembly mechanisms with different rates. On the basis of these observations, we proposed a min-max model that could account for the vast flagellar length range observed for asynchronous *L. pyrrhocoris.* This model may also apply to other flagellated organisms with flagellar length variation.

## Abstract (precis)

Current understanding of flagellum biogenesis during the cell cycle in trypanosomatids is limited to a few pathogenic species, including Trypanosoma brucei, Trypanosoma cruzi, and *Leishmania* spp. The most notable characteristics of trypanosomatid flagella studied so far are the extreme stability and lack of ciliary disassembly/absorption during the cell cycle. This is different from cilia in *Chlamydomonas* and mammalian cells, which undergo complete absorption prior to cell cycle initiation. In this study, we examined flagellum duplication during the cell cycle of *Leptomonas pyrrhocoris*. With the shortest duplication time documented for all Trypanosomatidae and its amenability to culture on agarose gel with limited mobility, we were able to image these cells through the cell cycle. Rapid, cell cycle-specific flagellum disassembly different from turnover was observed for the first time in trypanosomatids. Given the observed length-dependent growth rate and the presence of different disassembly mechanisms, we proposed a min-max model that can account for the flagellar length variation observed in *L. pyrrhocoris*.

## OBSERVATION

Recent comparative genomic analyses of Leptomonas pyrrhocoris, a monoxenous parasite (one host in the life cycle), provide profound insights into the evolution of parasitism, particularly compared to closely related dixenous (two hosts in the life cycle) *Leishmania* spp. (1). The presence of a full set of genes responsible for RNA interference also makes L. pyrrhocoris a promising model for functional studies. In this study, we examined flagellum duplication during the *L. pyrrhocoris* cell cycle, considering its importance in trypanosomatid physiology and pathogenesis (2).

*L. pyrrhocoris* proliferated robustly in Schneider Drosophila medium. The cells replicated every 4.2 ± 0.2 h, with exponential growth observed over a range of 2 × 10^4^ to 2 × 10^7^ cells/ml (see Fig. S1 in the supplemental material). The *L. pyrrhocoris* cell cycle is the shortest reported for trypanosomatids that we are aware of, compared to 8 to 10 h for Trypanosoma brucei, ∼7 h for Leishmania mexicana, ∼10 h for Leishmania major, ∼12 h for Trypanosoma lewisi, ∼25 h for Trypanosoma cruzi, and ∼7 h for Crithidia fasciculata. Of note, the vast majority of monoxenous species have not been investigated in this regard (3).

10.1128/mBio.02424-19.1 FIG S1 Rapid proliferation of *L. pyrrhocoris* in Schneider Drosophila medium (SDM) culture. *L. pyrrhocoris* was cultivated in SDM supplemented with 10% heat-inactivated fetal calf serum at 28°C. A fresh culture was inoculated at a density of 2 × 10^4^ cells/ml. (A) Cell density was monitored using a hemocytometer in the same culture without further dilution for 48 h. (B) Duplication number at each time point was calculated as log_2_ (cell density/2 × 10^4^). Exponential proliferation was modelled by linear regression. Download FIG S1, PDF file, 0.1 MB. Copyright © 2019 He et al. 2019 He et al. This content is distributed under the terms of the Creative Commons Attribution 4.0 International license.

**FIG 1:**
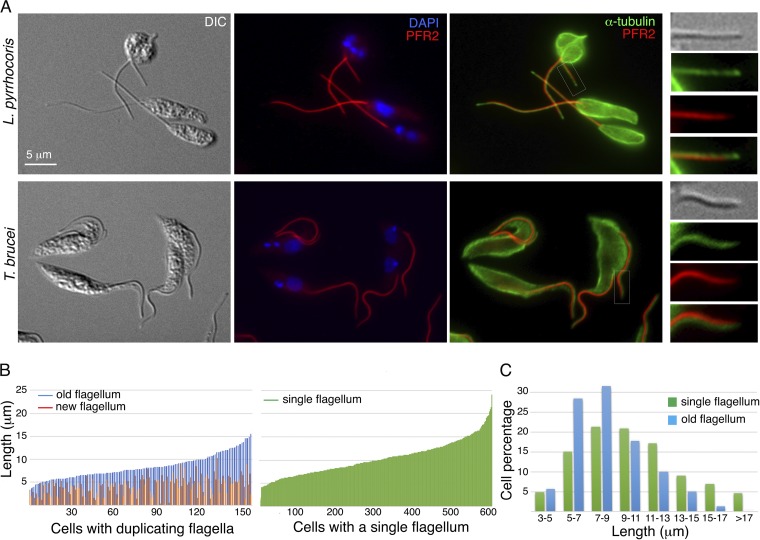
Length variation of *L. pyrrhocoris* flagella. *L. pyrrhocoris* cells were fixed and stained with anti-α-tubulin and anti-PFR2 antibodies, which label the microtubules and the paraflagellar rod, respectively. (A) The *L. pyrrhocoris* flagellar staining pattern was compared with that of T. brucei tse-tse infective, procyclic cells. Insets show enlarged views of the boxed regions. DIC, differential interference contrast; DAPI, 4′,6′-diamidino-2-phenylindole. (B) Using PFR2 stain as a flagellum marker, flagellar length was measured in 158 cells containing duplicating flagella and 604 cells containing a single flagellum. The cells are shown in order of increasing length of the old or single flagellum in the cell. (C) Length variation of the single flagellum and the old flagellum in duplicating cells.

To analyze the flagellum, *L. pyrrhocoris* cells were labeled with antibodies against PFR2, which is a highly conserved component of the paraflagellar rod (PFR) present along the trypanosomatid axoneme (4), and anti-α-tubulin antibodies that mark the axoneme microtubules (Fig. 1A). The PFR in T. brucei is found along the axoneme, ending at the same distal point as the axoneme microtubules. In *L. pyrrhocoris*, however, a gap between the PFR and axoneme tip was consistently observed. Similar observations were also made with antibodies against PFR1, another conserved PFR component (Fig. S2). In trypanosomes, the PFR has been proposed to act as a structural framework to mediate flagellar signaling events (5) and regulate axonemal waveform (6, 7). The absence of PFR at the distal tip of *L. pyrrhocoris* flagella implies a different signaling mechanism or motility modulation at the flagellar tip in this parasite.

10.1128/mBio.02424-19.2 FIG S2 Distinct flagellar tip organization in T. brucei and *L. pyrrhocoris. L. pyrrhocoris* and procyclic T. brucei were fixed with methanol at –20°C and stained with anti-alpha-tubulin and anti-PFR1 antibodies, which label the microtubules and the paraflagellar rod, respectively. Insets show enlarged views of the boxed regions. Download FIG S2, PDF file, 0.7 MB. Copyright © 2019 He et al. 2019 He et al. This content is distributed under the terms of the Creative Commons Attribution 4.0 International license.

In addition to the flagellar axoneme, the anti-α-tubulin antibodies also label the subpellicular microtubules that demarcate the trypanosomatid cell bodies. *L. pyrrhocoris* cells with a single flagellum are mostly rod shaped, but replicating cells with two flagella are more rounded (Fig. 1A), similar to the previously reported cases of L. mexicana and L. major (8, 9). Such morphological remodeling of the mother cell was not observed during the T. brucei cell cycle. When *L. pyrrhocoris* was stained with YL1/2, a monoclonal antibody directed against tyrosinated tubulin in newly synthesized microtubules (10), increased staining was observed in replicating cells (Fig. S3), suggesting increased subpellicular microtubule turnover in cells that were producing a new flagellum.

10.1128/mBio.02424-19.3 FIG S3 Morphological remodelling and increased microtubule turnover in replicating *L. pyrrhocoris. L. pyrrhocoris* was fixed with cold methanol and stained with YL1/2 and anti-PFR2 antibodies, which labeled tyrosinated microtubule and paraflagellar rod, respectively. Increased YL1/2 staining was observed in cells with replicating flagella, marked by asterisks. YL1/2 staining was also consistently detected at the tip of the nascent flagellum (arrows), while little YL1/2 staining was found in old flagella. Enlarged views of the boxed regions are shown in the lower panels. Download FIG S3, PDF file, 0.5 MB. Copyright © 2019 He et al. 2019 He et al. This content is distributed under the terms of the Creative Commons Attribution 4.0 International license.

Flagellar length (marked by anti-PFR2) was measured in a log-phase, asynchronous population of *L. pyrrhocoris* cells. A total of 762 cells were measured, with 604 cells containing a single flagellum and 158 cells with duplicating flagella (Fig. 1B). Similar to L. mexicana, but unlike T. brucei, *L. pyrrhocoris* displayed a vast variation in flagellar length. In cells containing a single flagellum, the flagellar length varied between 3 and 24 μm, with ∼10% of the flagella longer than 15 μm and ∼35% longer than 10 μm. Length variation (3 to 17 μm) was also observed for the longer, old flagellum in replicating cells. However, only 1.3% of the old flagella were longer than 15 μm and 21.5% were longer than 10 μm. This length variation is consistent with continuous flagellum growth after cell division, as has been previously reported for T. brucei and L. mexicana (8, 11). It also raises an intriguing possibility that the existing old *L. pyrrhocoris* flagellum undergoes partial disassembly at the time of new flagellum biogenesis. Flagellum disassembly/absorption is well documented in green algae and mammalian cells, where complete disassembly occurs prior to cell division (12, 13). In trypanosomatids, flagellar disassembly has been observed during the differentiation (14, 15), but not during the cell cycle (16).

**FIG 2:**
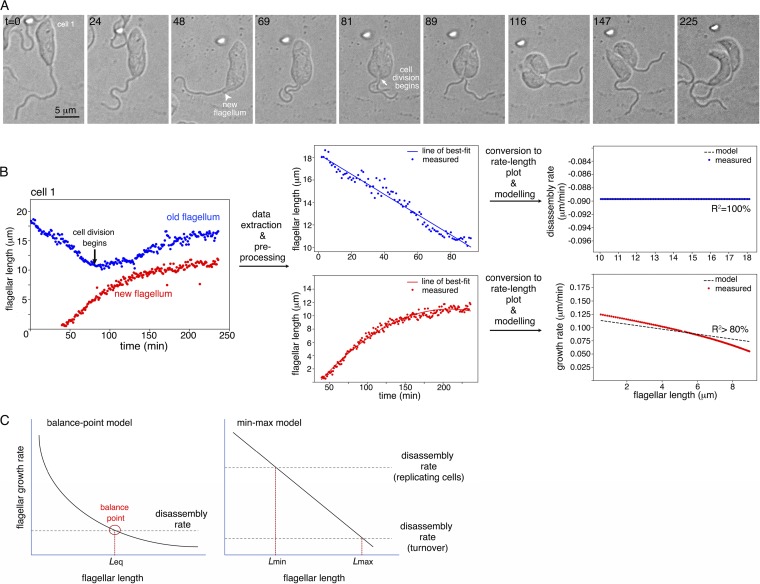
Flagellum length dynamics in dividing *L. pyrrhocoris.* (A) Differential interference contrast images of live *L. pyrrhocoris* cells were acquired every 1 min for ∼4 h. Selected time frames with key morphological events are shown. Numbers in the top left corners represent time in minutes. The complete movie sequence can be found in Movie S1 in the supplemental material. (B) The length of both the old flagellum and new flagellum was measured over the entire time lapse. Flagellum disassembly was modelled using measurements on the old flagellum, and flagellum net growth was modelled using measurements from the new flagellum. The workflow demonstrates length measurements, data extraction, plot conversion, and modeling steps on one representative cell shown in panel A. (C) Comparison of the balance point model and the min-max model derived from analyses of *L. pyrrhocoris*. In the balance point model, the flagellum disassembles at a constant rate, while the flagellum assembly rate is inversely proportional to flagellar length. When the assembly rate equals the disassembly rate (balance point), an equilibrium flagellum length (Leq) is achieved. In *L. pyrrhocoris*, the flagellum growth rate is found to decrease with flagellar length, with a near constant deceleration. Flagellum disassembly appears regulated, with faster disassembly observed during new flagellum biogenesis and slower turnover at other times. These two different, cell cycle-specific disassembly rates thus define the minimum and maximum length of the flagellum and account for the large length variation observed in *L. pyrrhocoris*.

To visualize flagellum length dynamics during the cell cycle, partially immobilized *L. pyrrhocoris* cells were imaged every 1 min for ∼4 h (see Text S1 in the supplemental material). Flagellar length was measured in five cells that underwent flagellum duplication and cell division during the imaging time (Fig. 2; see also Fig. S4 and Movie S1 in the supplemental material). In all cells, shortening of the old flagellum was observed prior to the emergence of a new flagellum protruding from the cell body. As the new flagellum was assembled, the old flagellum continued to shorten until cell division began. Flagellar shortening ended, and old flagellum resumed growth only after cell division was completed. Best-fit models of the disassembly process in the old flagellum and the growth phase in the new flagellum were generated separately using linear regression (Fig. 2B; Text S1).

10.1128/mBio.02424-19.4 FIG S4 Flagellar length measurements for cell 2 and cell 3. The length of both the old flagellum and the new flagellum in cell 2 and the single flagellum in cell 3 was measured over the entire time lapse shown in Movie S1. Download FIG S4, PDF file, 0.5 MB. Copyright © 2019 He et al. 2019 He et al. This content is distributed under the terms of the Creative Commons Attribution 4.0 International license.

10.1128/mBio.02424-19.5 MOVIE S1 Time lapse imaging of live *L. pyrrhocoris* cells. Partially immobilized *L. pyrrhocoris* cells were imaged every minute for 4 h on an inverted microscope equipped with a 60× objective with a numerical aperture (NA) of 1.4. In this video, two cells (cell 1 and cell 2) underwent flagellum duplication and cell division during the imaging period. Continuous flagellar growth was observed in cell 3. Flagellar length measurement of cell 1 is shown in Fig. 2. Measurements of cell 2 and cell 3 are shown in Fig. S4. Download Movie S1, MOV file, 16.8 MB. Copyright © 2019 He et al. 2019 He et al. This content is distributed under the terms of the Creative Commons Attribution 4.0 International license.

10.1128/mBio.02424-19.6 TEXT S1 Supplemental methods. Download Text S1, PDF file, 0.04 MB. Copyright © 2019 He et al. 2019 He et al. This content is distributed under the terms of the Creative Commons Attribution 4.0 International license.

The flagellar disassembly rate was found to be highly constant throughout the shortening phase in different *L. pyrrhocoris* cells at ∼0.089 μm/min (*R*^2^ = 100%, root mean square error [RMSE] = 0). This rate is lower than the absorption rate of 0.31 ± 0.07 μm/min reported for *Chlamydomonas* flagella (17), but nearly 20 times higher than a turnover rate at ∼0.28 μm/h (i.e., ∼0.0047 μm/min) predicted by previous modeling of L. mexicana (8). *L. pyrrhocoris* flagella grew at various rates following an inverse relationship to flagellar length, which can be described as follows: *dL*/*dT* = *A* − *BL*, where *dL*/*dT* is the rate of net growth, and *L* is flagellar length. By averaging the best-fit models from four of the five cells (one was discarded due to poor *R*^2^ value), we found constant *A* = 0.108 μm/min, which represents the theoretical maximum growth rate when *L* = 0, and the flagellum assembly rate constant *B* = 0.004/min.

Eukaryotic flagellar length regulation has been best studied in cells with relatively fixed flagellar length. As flagellar components are constantly added and removed at the distal tip, flagellar length is the balanced result of both assembly and disassembly processes. In Chlamydomonas reinhardtii, flagellum disassembly has been found to be constant and length independent. Assembly, however, is inversely proportional to length (Fig. 2C). At the balance point, where the assembly rate equals the disassembly rate, an equilibrium flagellar length (Leq) is achieved. How flagellar growth rate decreases over length is not fully understood, and several different mechanisms have been proposed to explain this phenomenon (17–20). In T. brucei, mature flagellum is highly stable. A grow-and-lock model posits that a lock mechanism takes effect around the time of cell division, preventing the newly synthesized flagellum from further growth or shortening (16).

However, not all flagellated organisms have flagella of uniform length. Flagellar length variation in L. mexicana was explained as a result of continuous elongation throughout several cell cycles. Flagellar turnover was also predicted by previous modeling of L. mexicana (8). However, flagellum shortening was not directly observed in live-cell imaging of L. mexicana, likely due to its long(er) cell cycle. In *L. pyrrhocoris*, the old flagellum disassembled at a constant rate of ∼0.089 μm/min at the time of new flagellum synthesis. For the new flagella, an inverse correlation was found between the assembly rate and flagellar length. Combining these two observations, we would expect a balance point with a flagellar length of ∼4μm (Fig. 2C), correlating with the shortest length measured for *L. pyrrhocoris* flagella (Fig. 1B). On the other hand, the longest flagellar length observed was ∼24 μm, corresponding to a disassembly rate of ∼0.012 μm/min that is ∼7 times lower than the observed ∼0.089 μm/min disassembly rate. To unify these observations, we proposed that *L. pyrrhocoris* has two distinct disassembly mechanisms, one at ∼0.089 μm/min that initiates around the time of new flagellum synthesis and ends at cytokinesis, and the other one at ∼0.012 μm/min and is a constitutive, cell cycle-independent turnover rate.

The observed length-dependent growth rate, together with the presence of two distinct disassembly processes, predict two possible balance points that correspond to the maximum and the minimum length a flagellum can achieve. We term this a min-max model (Fig. 2C). Rapid shortening of the old flagellum occurs during new flagellum biogenesis. After cell division, the disassembly rate decreases to the turnover rate, allowing both the old flagellum and the newly assembled flagellum to continue to grow in their respective daughter cells (e.g., cell 3 in Movie S1 and Fig. S4), and some reach maximum length before the initiation of the next cell cycle.

The min-max model could explain flagellar length variation in *L. pyrrhocoris* and perhaps also other organisms. Disassembly mechanisms involving evolutionarily conserved molecules and pathways have been reported (21–23). Homologs to key molecules, such as kinesin 13, have also been found in the *L. pyrrhocoris* genome. With further development of molecular genetic tools, the fast-replicating *L. pyrrhocoris* would be a useful model to understand flagellar length regulation in organisms containing flagella of variable lengths. It is generally accepted that flagellum length is important for its functions (24). Whether shortening of the old flagellum is required for new flagellum assembly calls for further understanding of the expression and regulation of flagellar components in *L. pyrrhocoris*. How swimming behavior of *L. pyrrhocoris* cells might be affected by different flagellar lengths and how the cells cope with such differences during the cell cycle also remain to be investigated.
