# Supplementary figures and images for: Cell Cycle-Dependent Flagellar Disassembly in a Firebug Trypanosomatid Leptomonas pyrrhocoris
Source: mBio. 2019 Nov 26;10(6):e02424-19. doi: 10.1128/mBio.02424-19 (PMC6879719; doi:10.1128/mBio.02424-19)

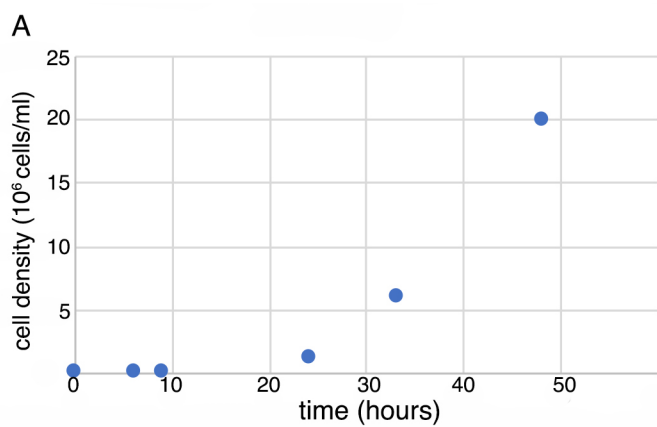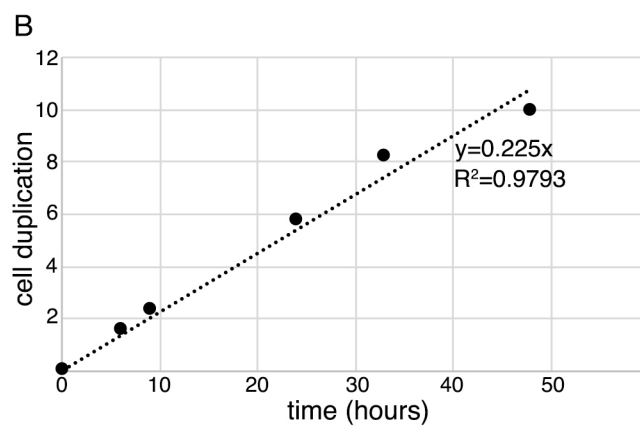

Supplement: FIG S1 [file mBio.02424-19-sf001.pdf]

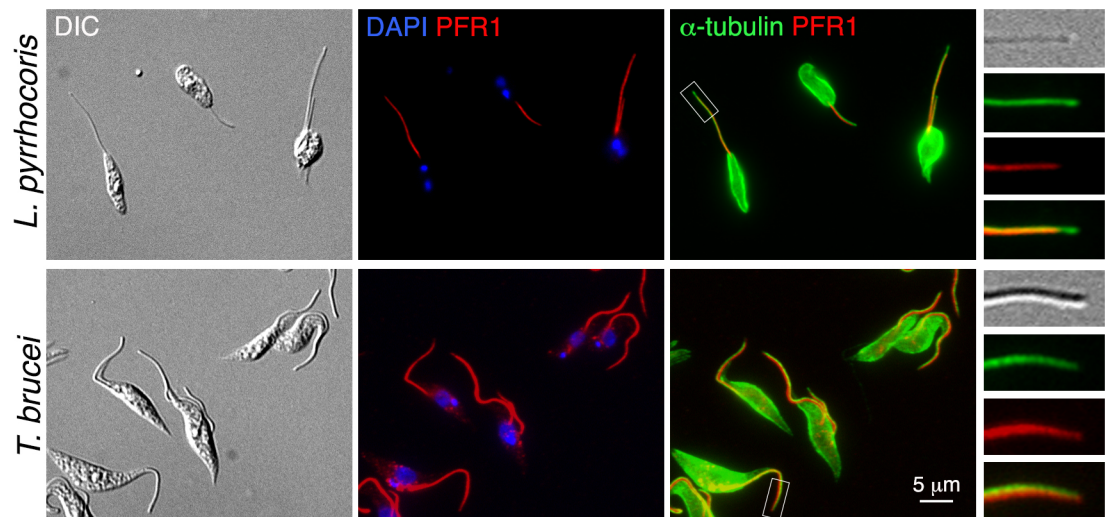

Supplement: FIG S2 [file mBio.02424-19-sf002.pdf]

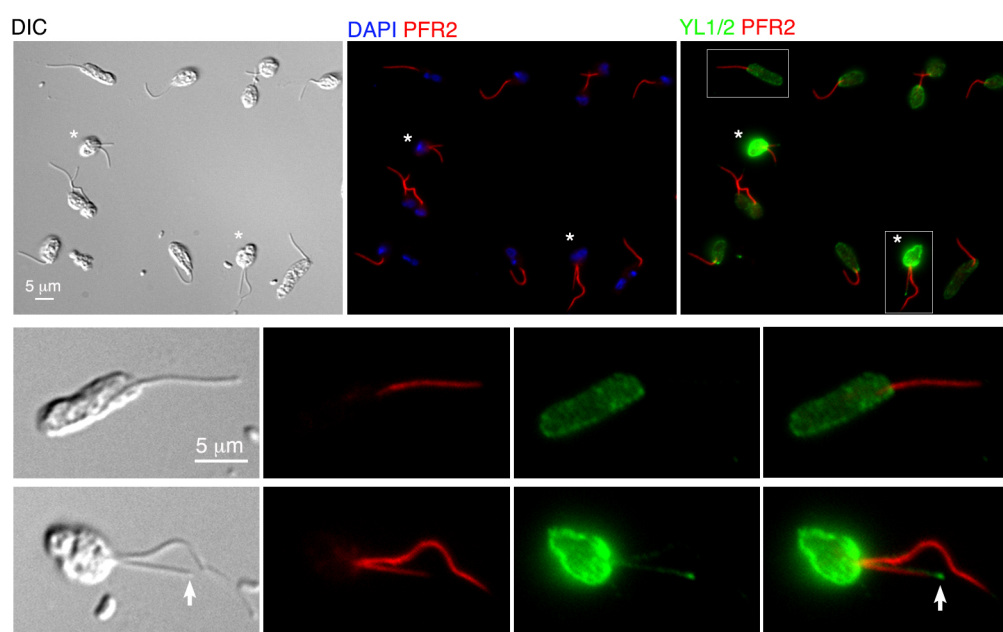

Supplement: FIG S3 [file mBio.02424-19-sf003.pdf]

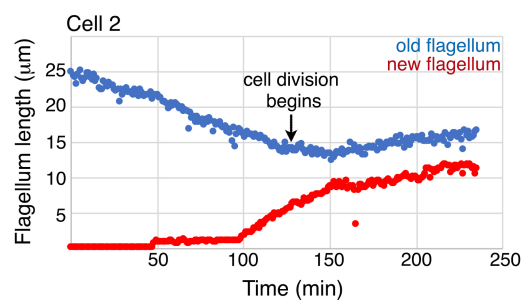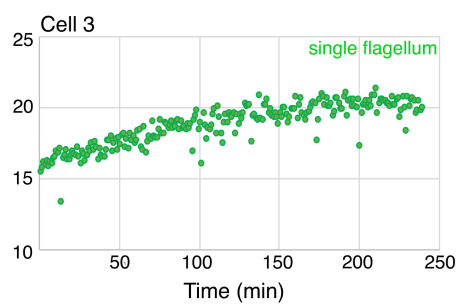

Supplement: FIG S4 [file mBio.02424-19-sf004.pdf]
