# Supplementary material for: Cell Cycle-Dependent Flagellar Disassembly in a Firebug Trypanosomatid Leptomonas pyrrhocoris
Source: mBio. 2019 Nov 26;10(6):e02424-19. doi: 10.1128/mBio.02424-19 (PMC6879719; doi:10.1128/mBio.02424-19)
Supplement: TEXT S1 [file mBio.02424-19-s0001.pdf]

Additional methods

Live cell imaging of partially immobilized L. pyrrhocoris

The live cell imaging was performed according to previously described method (1) with minor modifications. To prepare the agarose gel, 0.5 g low melting agrose (Gibco, Thermo Fisher Scientific, Waltham, USA) and 0.5 g electrophoresis-grade agarose (Invitrogen, Thermo Fisher Scientific) were mixed with 9 ml dH_2O, heated in a microwave and then transferred to a water bath at 55°C. 90 ml SDM (Gibco, Thermo Fisher Scientific) and 10 ml heat-inactivated fetal calf serum (HyClone, GE Healthcare Life Sciences, Pittsburgh, USA) were added slowly to the melted agarose with constant, gentle mixing. The agarose mixture was immediately poured into 5cm petri dishes (4 ml each dish) and allowed to set at room temperature. The agarose plates were air dried in a tissue culture hood for 10 min before use. Unused plates were stored at 4°C and brought to room temperature before use. 200-300μl log phase *L. pyrrhocoris* culture was added to the plate and spread evenly on the surface. A ~1.5 cm × 1.5 cm gel slice was excised and inverted onto a 35mm μ-Dish with glass bottom (ibidi GmbH, Gräfelfing, Germany). The cells were thus trapped and partially immobilized between the agarose gel and the cover glass bottom.

Time lapse imaging was conducted on an Axiovert inverted microscope (Carl Zeiss AG, Oberkochen, Germany) equipped with a 60× NA1.4 objective and a CoolSNAP HQ2 (Teledyne Photometrics, Tuscon, USA) camera. Differential interference contrast (DIC) images were acquired every 1 min for 4 hours. Images were analyzed using measurement tools in ImageJ (NIH). Figures and Movie 1 were prepared using ImageJ and Photoshop (Adobe Inc., San Jose, USA).

Mathematic modelling methods

Flagellar length modelling was performed using custom scripts in Python (see below). Dataset from a total of 5 cells that underwent flagellar duplication and cell division during the 4-hour imaging period, were used for model training and testing. For both old and new flagella, flagellum length vs. time was plotted. After removing outliers and missing values (which can lead to false slope), best-fit models (R^2 > 95%) with smoothened curves were generated for each dataset. The approximate assembly and disassembly rate was calculated using differential method, and plotted against flagellar length.

We first tried to simulate our dataset based on the well-established balance point model (2). Using GEKKO, a package in Python (Script A), we tried to find out the value of constants P and L in equation: dL/dt = P/L – Q, where L is flagellar length and dL/dt is the flagellar assembly rate. No positive values of P and Q could be obtained, suggesting that flagellar length regulation in *L. pyrrhocoris* does not follow the balance point model. We therefore focused on developing another model to account for our observations.

Flagellum disassembly was modelled using data derived from the shortening phase of the old flagella; and flagellum assembly was modelled using data from the new flagella (as shown in Fig. 2B). After conversion to rate vs. length plots, best-fit models were developed between rate and length ($R^2 \approx 100\%$ for old flagella and R^2 > 85% for new flagella) using linear regression (Script B).

<u>Script A</u>

```
import numpy as np
from gekko import GEKKO
import matplotlib.pyplot as plt
import pandas as pd
import math
import scipy.stats as stats
from sklearn.metrics import mean_squared_error, r2_score
import xlsxwriter
from sklearn import preprocessing
# Importing the dataset
dataset1 = pd.read_csv('c_a.csv')
dataset = dataset1.dropna(subset=['A_rate'])
```

```
xm = dataset.iloc[:, 2:3].values
ym = dataset.iloc[:, 3].values

# define GEKKO model
m = GEKKO(remote=False)
# parameters and variables
a = m.FV(value=0)
b = m.FV(value=0)
c = m.FV(value=0,lb=-100,ub=100)
x = m.Param(value=xm)
ymeas = m.Param(value=ym)
ypred = m.Var()
# parameter and variable options
a.STATUS = 1 # available to optimizer
b.STATUS = 1 #  to minimize objective
c.STATUS = 1
# equation
m.Equation(ypred == b*26.3 - b*x)
# objective
m.Obj((ypred-ymeas)**2/len(ymeas))
# application options
m.options.IMODE = 2   # regression mode
# solve
m.solve() # remote=False for local solve
#m.open_folder()
# show final objective
print('Final SSE Objective: ' + str(m.options.objfcnval))

# print solution
print('Solution')
print('a = ' + str(a.value[0]))
print('b = ' + str(b.value[0]))
print('c = ' + str(c.value[0]))
```

Script B

```
# Importing the libraries
import numpy as np
import matplotlib.pyplot as plt
import pandas as pd
import math
import scipy.stats as stats
from sklearn.metrics import mean_squared_error, r2_score
import xlsxwriter
from sklearn import preprocessing
# Importing the dataset
dataset = pd.read_csv('c_a.csv')
#dataset = dataset1.dropna(subset=['A_rate'])
X = dataset.iloc[:, 0:1].values
y = dataset.iloc[:, 1].values

# Fitting Polynomial Regression to the dataset
from sklearn.linear_model import LinearRegression
from sklearn.preprocessing import PolynomialFeatures
poly_reg = PolynomialFeatures(degree = 2)
X_poly = poly_reg.fit_transform(X)
poly_reg.fit(X, y)
lin_reg_2 = LinearRegression()
lin_reg_2.fit(X_poly, y)
```

```
y_predicted = lin_reg_2.predict(poly_reg.fit_transform(X))

#Printing the equation
coefficient = lin_reg_2.coef_
intercept = lin_reg_2.intercept_
print("Equation is:")
print("y = ", end='')
print(intercept,"+ ",end='')
for i in range(1,len(coefficient)):
    print(coefficient[i],"x^",end='')
    print(i,end=' ')
    if i == len(coefficient)-1:
        break
    else:
        print('+',end=' ')

# evaluating the model on test dataset
rmse_dataset = np.sqrt(mean_squared_error(y, y_predicted))
r2_dataset = r2_score(y, y_predicted)
#Printing RMSE and R2 score
print("\nThe model performance for the given data set")
print("RMSE is {}".format(rmse_dataset))
print("R2 score is {}".format(r2_dataset))

#Visualizing the data and model
plt.scatter(X, y, color = 'red', s=4, label = 'bx')
plt.plot(X, y_predicted, color = 'blue', label = 'ro')
plt.xlabel('time (in minutes)')
plt.ylabel('length (in µm)')
plt.legend(['Predicted','Measured'],loc='best')
plt.savefig("NewLenvstime/cellc.png", dpi=200, bbox_inches='tight')
plt.show()
```

References

1. He CY, Ho HH, Malsam J, Chalouni C, West CM, Ullu E, Toomre D, Warren G. 2004. Golgi duplication in *Trypanosoma brucei*. J Cell Biol 165:313-21.
2. Marshall WF, Qin HM, Brenni MR, Rosenbaum JL. 2005. Flagellar length control system: testing a simple model based on intraflagellar transport and turnover. Mol Biol Cell 16:270-278.
